# Biological Knowledge of Thornback Ray (*Raja clavata*) from the Azores: Improving Scientific Information for the Effectiveness of Species-Specific Management Measures

**DOI:** 10.3390/biology10070676

**Published:** 2021-07-17

**Authors:** Régis Santos, Wendell Medeiros-Leal, Ana Novoa-Pabon, Osman Crespo, Mário Pinho

**Affiliations:** 1 IMAR Institute of Marine Research, University of the Azores, 9901-862 Horta, Portugal; wendell.mm.silva@uac.pt (W.M.-L.); mario.rr.pinho@uac.pt (M.P.); 2 Okeanos R&D Centre, University of the Azores, 9901-862 Horta, Portugal; ana.mn.pabon@uac.pt (A.N.-P.); osman.cresponeto@imbrsea.eu (O.C.); 3 Department Oceanography and Fisheries, Faculty of Science and Technology, University of the Azores, 9901-862 Horta, Portugal

**Keywords:** elasmobranchs, skates, demersal, commercial fish, life history, stock structure, assessment, fisheries management

## Abstract

**Simple Summary:**

Sharks, rays, and skates are increasingly being recognized as endangered due to their life-history characteristics, fishing pressure, and habitat degradation. The thornback ray *Raja clavata* is one of the most commercially important skates in the seas of Northwest Europe. However, due to a lack of biological knowledge about this species in Azorean waters, the types of stock evaluations that can be performed are restricted. This study expands current knowledge on vertical distribution, size-frequency distributions, growth patterns, sex ratios, mortality rates, and reproduction of this species, and provides a baseline for further fishing monitoring.

**Abstract:**

Elasmobranchs are globally recognized as vulnerable due to their life-history characteristics, fishing pressure, and habitat degradation. Among the skates and rays caught by commercial fisheries, the thornback ray *Raja clavata* is one of the most economically important in Northwest European seas. However, the scarcity of biological knowledge about this species in Azorean waters has limited the stock assessment types that can be conducted. To improve information on its habitat preferences, spatial distribution and movement pattern, growth, sex ratio, mortality, and reproduction, as well as to investigate long-term changes in abundance and size, this study analyzed approximately 25 years of fishery-dependent and independent data from the Azores. *Raja clavata* was mainly caught at depths up to 250 m. Most of the tagged fish were recaptured near the release point. A larger–deeper trend was found, and females were larger and more abundant than males. Life-history parameters showed that *R. clavata* has a long lifespan, large size, slow growth, and low natural mortality. The sustainability of its population is of concern to fisheries management and, while our findings suggested a relatively healthy stock in the Azores, a thorough increase in data quality is required to better understand the stock condition and prevent overexploitation.

## 1. Introduction

Elasmobranchs (sharks, skates, and rays) are widely recognized as a fragile resource, more susceptible to decline and extinction than most teleost fishes, due to their life-history characteristics (low fecundity, late maturity, and slow growth rates), fishing pressure, and habitat degradation [1,2]. Sharks, skates, and rays are often caught as bycatch by commercial fisheries but are often retained on board because of the high, and in some cases rising, value of their meat, fins, and livers [2,3,4]. Since most captures are uncontrolled and often misidentified, unrecorded, aggregated, or discarded at sea, there is a scarcity of species-specific landing data [2,4,5]. At the same time, there is a lack of biological knowledge, such as life history, habitat use, movement patterns, and population structure, for most species. Such characteristics may have serious implications for the sustainability of elasmobranch fisheries, as science-based measures are important to avert population collapse [6].

Among the skates and rays frequently captured by the commercial fishery, the thornback ray *Raja clavata* Linnaeus, 1758 (Chondrichthyes: Rajidae) is one of the most important species in the seas of Northwest Europe [7,8,9]. Global total catches in 2019 were 6874 t, with the highest catches recorded in France (1876 t), the United Kingdom (1372 t), Portugal (868 t), and Italy (838 t) [10]. *Raja clavata* is a widely distributed skate in the Eastern Atlantic and Southwest Indian Ocean, including the North Sea, the Macaronesian archipelagos (the Azores, Madeira, Canaries), the Mediterranean Sea, and the Western Black Sea [11]. It inhabits shelf and slope waters on mud-to-sand substrates at depths between 5 and 1020 m, but is usually found in shallow waters up to 250 m [12]. Spawning appears to happen mostly during the summer (regardless of latitude or water temperature), although the length of the whole spawning season seems to be prolonged in warmer sea temperatures [13].

In the Azores, genetic studies support the existence of a self-contained *R. clavata* population, i.e., a stock unit for which it is assumed that abundance dynamics are determined by internal processes of recruitment and mortality, and insignificantly affected by immigration and emigration [14,15]. *Raja clavata* in the Azorean region represents more than 90% of the landed skates [16], and it is mainly caught by the demersal fishery using hook and lines [17]. Catch trends of this species have declined steeply from 171 t (5.6 kg 10^−3^ hooks) in 2015 to 70 t (3.1 kg 10^−3^ hooks) in 2017 [9]. No information on the discard rates of skates is available for recent years. Nevertheless, discarding is known to take place, and is the result of management measures, particularly the total allowable catches—TACs/quotas, minimum size, and fishing area restrictions rather than the complete lack of a market [9,16,18]. On the other hand, previous studies have reported that the discard survival of skate species is high [19,20,21,22], particularly for *R. clavata* caught by longline fishing [9,23].

Currently, the International Council for the Exploration of the Sea (ICES) assesses the *R. clavata* from the Azores based on a precautionary approach because of the data quality (ICES stock category 3; [16]). There is poor knowledge of the biology of the species for this ecoregion and available information is uncertain. The abundance index derived from the Azorean bottom longline survey [24] is used as an index of stock development; however, stock status relative to reference points is unknown [25]. Considering this, the ICES working group on elasmobranch fishes (WGEF) recommends that further studies should be conducted to define the appropriate set of life-history parameters and describe the population dynamics in order to provide more accurate data for exploratory assessments [16].

In this context, this study aimed to analyze information on distribution and movement patterns, life-history aspects (sex ratio, reproductive season, size at maturity, growth parameters, mortality rates), size composition, and abundance of the thornback ray *R. clavata* derived from scientific surveys and commercial fisheries in the Azores region. Fishing-induced changes in abundance and size structure and preliminary exploitation status were also investigated. Findings from this study are expected to facilitate stock assessment and support reliable fishery management strategies.

## 2. Material and Methods

### 2.1. Data Collection

Data analyzed in this study were derived from scientific surveys, commercial catches, and official commercial landings in the Azores region (ICES Subdivision 27.10.a.2).

#### 2.1.1. Scientific Surveys

Spring bottom longline surveys were performed from 1996 to 2019 around the islands and major seamounts of the Azores archipelago. The survey followed a stratified random sampling design in which each sampling area was divided into depth strata with 50 m intervals down to 1200 m depth. Each bottom longline set was deployed perpendicular to the isobaths. Catches per unit of effort were weighted by the corresponding area size to estimate the relative abundance indices (relative population number—RPN; ind. 10^−3^ hooks). 

Total length (*L_T_*) and sex was recorded for each captured skate. Of these, 2351 individuals were marked with a numbered plastic tag (Hallprint Fish Tags, Hindmarsh Valley, Australia) and released at the sea surface. After being released, the fish condition (active, less active, or inactive) was documented, and the geographic position was registered with a GPS. 

The macroscopic maturity stage was determined for a sample of 381 individuals captured between 1996 and 2013. 

Further details on survey design and abundance estimates can be found in Pinho et al. [24].

#### 2.1.2. Commercial Catches

Commercial catch data were collected within the European Commission’s data collection framework (DCF) [26] during the period 1990–2017. Structured inquiries (*n* = 31,616) were conducted with the vessels’ captains of the local fleet during their landings at Azorean ports. Each inquiry included the vessel ID and size, departure and arrival dates, fishing gear type, average depth zone of the fishing operation, and catch in weight by species. 

Biological information (*L_T_*, sex, maturity stage, gonadosomatic and hepatosomatic indices) was taken for 390 individuals caught throughout the year by the Azorean commercial fleet between 2005 and 2017. 

DCF sampling design and protocols were aligned with the recommendations of the ICES working groups on commercial catches (WGCATCH) and biological parameters (WGBIOP) [27].

#### 2.1.3. Official Commercial Landings

Official landings (in tons) were obtained from the Azores Auction Services (Lotaçor S.A.) for the period 1990–2020. 

Information on *L_T_* for combined sexes was available for a sample (*n* = 18,181) of thornback rays landed until 2017. 

### 2.2. Data Analyses

#### 2.2.1. Distribution

To describe the relationships between presence–absence and survey-derived abundance indices of the thornback ray *R. clavata* and habitat characteristics, generalized additive models (GAMs) [28,29] were implemented with the *mgcv* package [30,31,32,33,34] in R, version 4.0.3 [35]. Due to the large proportion of zero values in the RPN data (92%), the presence–absence data were fitted separately, using a binomial error distribution and logit link function, from the positive abundances, which were fitted using a Gaussian error distribution with identity link function [36,37]. This approach has been shown to work well with zero-inflated data [37,38]. Explanatory variables included in the analyses were latitude and longitude (as an interaction term), depth, and substrate type. Species distributional data based on range maps (extent-of-occurrence) or survey data frequently exhibit spatial autocorrelation, which means that sites adjacent to each other have more comparable values than those further away [39]. Some efforts are made to address these problems, such as explicitly adding latitude and longitude as a smoothed interaction factor in GAMs [33]. While these issues are ignored when modeling fish distribution, they leads to a number of difficulties, including poor model fit and performance, skewed predictions, and high model sensitivity to parameter changes [39,40,41]. Geographical coordinates and nominal depth were obtained during the fishing gear deployment in the surveys. The bottom type was extracted from EMODnet seabed habitat compilations (www.emodnet-seabedhabitats.eu accessed on 9 March 2021) and categorized as mud (Mud), muddy sand (Mud.S), sandy mud (Sand.M), sand (Sand), mixed sediment (Mix.Sed), coarse sediment (C.Sed), or rock (Rock). Analysis of deviance results was used to indicate the explanatory variables that explained most of the variability in the RPN data. 

#### 2.2.2. Movement Patterns

Tagged skates were made available to be recaptured by the commercial fishery. A reward was offered to each fisherman providing tag–recapture information on *L_T_*, date, and geographical coordinates at which the fish was caught. Movement patterns were then assessed using the *marmap* R package. For this, a straight line was used to measure the traveled distance between capture and recapture geographical positions. 

#### 2.2.3. Size Structure

Size–frequency distributions observed in different regions (seamount and island) of the survey and those obtained from the official landings were examined for statistical similarity by applying a two-sample Kolmogorov–Smirnov (K–S) test. 

Differences in mean *L_T_* over the years and among depth strata (for survey data) were determined by Welch’s heteroscedastic *F* test and Bonferroni post hoc correction, using the *onewaytests* R package [42].

#### 2.2.4. Growth Parameters 

Growth parameters were estimated through the von Bertalanffy growth function (VBGF) [43] using monthly *L_T_*–frequency data (1-cm class interval) derived from the official landings for the period 2010–2016. As the *L_T_* data were not available for males and females separately, growth parameters were estimated for combined sexes. The asymptotic length (*L_∞_*), growth coefficient (*k*), and growth performance index (*Φ*) were calculated by electronical length–frequency analysis using a bootstrapped method with a genetic algorithm (ELEFAN_GA_boot; [44]) within the *TropFishR* [45,46] and *fishboot* [44,47] R packages. This analysis attempted to follow the best practices for using ELEFAN approach, such as, for example, a relatively high count that is representative of the *L_T_*–frequency distribution of the population or catches [45,47]. Bootstrapping involved 1000 resamples. 

Growth parameters were also estimated by analyzing tag–recapture data. To do this, *TropFishR* R package was used to build forced Gulland–Holt plots [48] and estimate *L_∞_* and *k* for combined and separated sexes.

#### 2.2.5. Sex Ratio

Proportions of males to females (M:F) by *L_T_*–class and depth stratum were compared with the expected 1:1 ratio using a chi-square test. 

#### 2.2.6. Reproduction

Information on the gonad maturity stage from the DCF database was insufficient or imprecise to estimate size at 50% maturity (*L_50_*). Thus, *L_50_* was estimated from the survey-derived *L_T_* data through logistic regression (Bayes) using the *sizeMat* R package [49]. Maturity stages for both sexes were classified into five phases (I—immature, II—developing, III—spawning capable, IV—actively spawning) adapted from Stehmann [50] and based on the macroscopic observation of the gonads. The regressing and regenerating stages were not adopted during sampling. Maturity stages III and IV were considered sexually mature. 

Although the monthly gonadosomatic index (GSI = gonad weight/total weight × 100) and hepatosomatic index (HSI = liver weight/total weight × 100) obtained from the commercial catches were available for both sexes, the constraints of the maturity stage classification did not allow us to exclude immature individuals for the reproductive seasonality analysis. The GSI and HSI data were, therefore, not analyzed in this study.

No information about fecundity was available.

#### 2.2.7. Mortality, Exploitation Rate, and Size at Capture

Mortality rates were calculated using the *L_T_* data taken from the official landings for the period 2010–2016. The total mortality rate (*Z*; year^−1^) was estimated based on the mean length data in the non-equilibrium situations method [51], and on the linearized length–converted catch curve [52]. The natural mortality (*M*; year^−1^) was computed as the average value of natural mortality estimated from different methods [53,54,55,56,57,58,59,60,61,62,63,64,65,66]. Fishing mortality (*F*; year^−1^) was obtained from the relationship between *Z* and *M*: *F* = *Z* − *M*. The exploitation rate (*E*) was determined by *E* = *F*/(*F + M*) [67]. The size at which 100% of individuals are vulnerable to capture (*L_c_*) was determined by using the peak of the *L_T_*–frequency distribution [51].

#### 2.2.8. Catch Rates and Landings

Interannual differences in RPN were examined by Welch’s test and Bonferroni post hoc correction. An unbiased yearly trend of catch per unit effort (CPUE; kg days at sea^−1^ vessel^−1^) derived from the commercial catches was provided and used for a trend comparison. It was estimated using a hurdle–lognormal generalized linear model (GLM) [36,68,69]. Year, quarter, vessel size, fishing gear, average depth zone of the fishing operation, and percentage of the capture of the thornback ray in relation to the total (target effect) were considered as potential drivers of CPUE. The GLM was run using the *lsmeans* R package [70]. Statistical details on this estimate are given by ICES WGEF [16].

Significance levels of all statistical analyses were set at a *p*-value of < 0.05.

## 3. Results

### 3.1. Distribution

**Figure 1:**
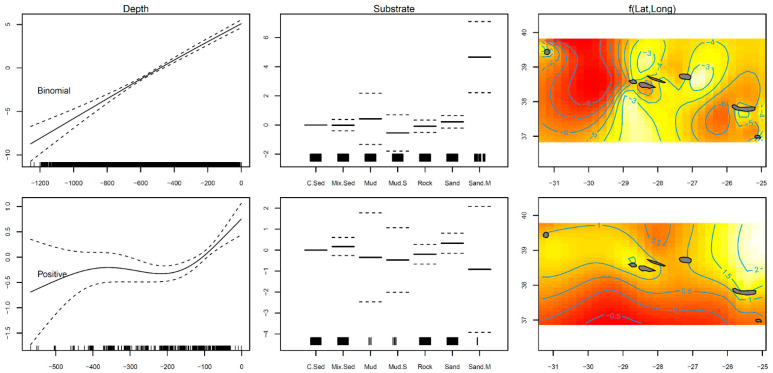
Residual plots for explanatory variables obtained by the presence–absence binomial and positive abundance Gaussian generalized additive models for the thornback ray *Raja clavata* caught during scientific surveys in the Azores. The smoother fit and ±0.95 confidence intervals are represented as solid and dashed lines, respectively. Tick marks on the x–axis indicate observed data points. White color indicates more individuals and red color fewer in the 2D smoother colored plot.

**Figure 2:**
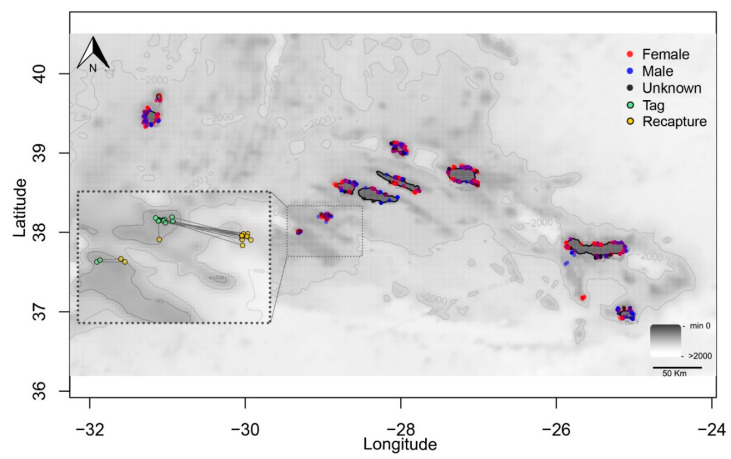
Tag release locations of female (red points), male (blue), and unsexed (black) thornback ray *Raja clavata* during scientific surveys (1996–2019) in the Azores. The green and yellow points indicate the release and recapture locations, respectively, of the 13 recaptured fish. Displacements between release and recapture points are represented by straight lines.

A total of 2846 individual thornback rays were sampled from the scientific surveys. The GAM results indicated that the presence–absence (binomial) model explained 37.9% of the variance, while the positive catches (Gaussian) model explained 16.3% (Table 1). The modeled data suggested a significantly greater presence of the thornback ray *R. clavata* on sandy mud habitats (*p* < 0.001; Table 1; Figure 1). Positive abundance was higher in coarse sediment bottoms (*p* < 0.001; Table 1; Figure 2). Latitude and longitude, as well as depth, were found to have a smoothing term significantly different from zero (*p* < 0.001) in fish presence and abundance; thus, they were relevant variables to the model’s fit (Table 1). The curve fitted to the modeled distribution revealed that the highest occurrence and abundance occurred in the depth range of 0–150 m, and at locations situated closer to the islands (Figure 1). 

### 3.2. Movement Patterns

After being released, 75% of the tagged skates were active, 14% were less active, and 1% were inactive. The fish condition was not reported for 10% of individuals. Only 35 (21 females, 7 males, and 7 unsexed; Table S1) of 2351 (1135 females, 940 males, and 276 unsexed) tagged skates were recaptured (recapture rate equals 1.5%). The mean time (±standard deviation [s.d.]) at liberty between tagging and recapture was 573.3 ± 506.0 days (min = 11 days, max = 1913 days). *L_T_* at capture ranged from 37.0 to 85.0 cm (mean = 61.7 cm, s.d. = 11.7), and ranged from 50.0 to 88.0 cm (mean = 69.6 cm, s.d. = 11.3) for skates that were later recaptured (Table S1). Twenty-two recaptured individuals were removed from the spatial analysis, as the geographic location at which they were recaptured was not properly recorded. Of the remaining 13 recaptured individuals, 23% were recaptured within 15 km of the release point, and 92% within 40 km (Figure 2). The distance traveled ranged from 9.1 to 40.5 km (mean = 30.7 km, s.d. = 11.6; Table S1).

### 3.3. Size Structure

**Figure 3:**
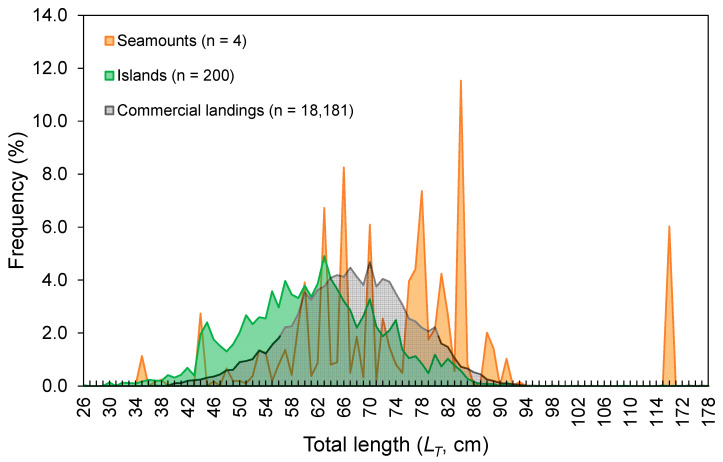
Total length (*L_T_*)–frequency distribution of the thornback ray *Raja clavata* derived from scientific surveys (1996–2019) and official commercial landings (1990–2017) in the Azores. For the surveys, data are shown separately for seamounts and islands, and the number of individuals (n) refers to the total RPN (ind. 10^−3^ hooks).

**Figure 4:**
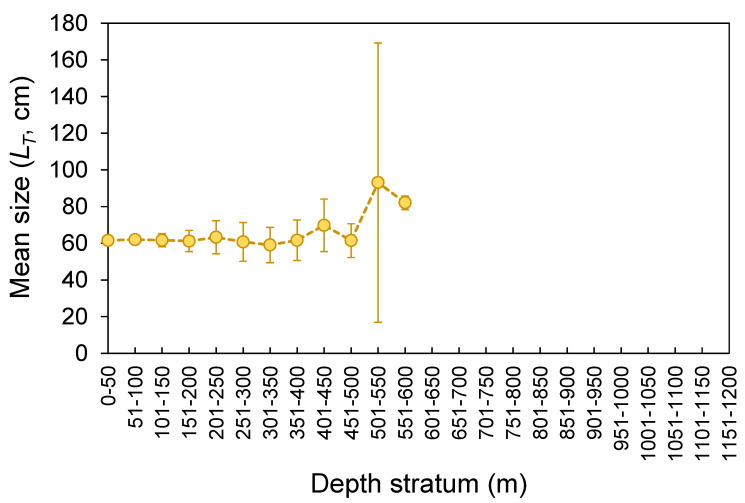
Depth distribution of mean (±0.95 confidence interval) total length (*L_T_*) of the thornback ray *Raja clavata* caught during scientific surveys (1996–2019) in the Azores.

**Figure 5:**
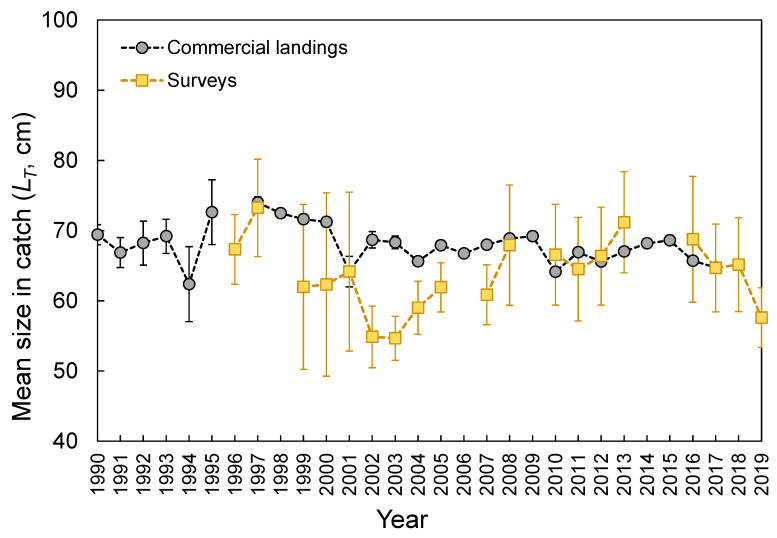
Annual mean (±0.95 confidence interval) of total length (*L_T_*) of the thornback ray *Raja clavata* caught during scientific surveys (1996–2019) and official commercial landings (1990–2017) in the Azores.

The *L_T_* ranged from 26 to 178 cm (Figure 3). No statistical differences were found between seamounts and islands (K-S test, *D* = 0.165, *p* = 0.143; Figure 3). The *L_T_* composition from the official commercial landings was similar to that observed from the scientific surveys (K-S test, *D* = 0.103, *p* = 0.681; Figure 3). However, the frequency of larger individuals (i.e., larger than 65 cm *L_T_*) was visually higher in commercial landing samples (Figure 3). A similar pattern could also be inferred for seamount; however, its sample size was very low and highly variable (Figure 3). Statistically significant differences (Welch’s ANOVA test, *F* = 97.3, *p* < 0.001) were observed in the mean sizes by depth, with larger individuals found between 500 and 600 m depths (Bonferroni correction post hoc test, *p* < 0.050; Figure 4). The *L_T_* information from the DCF dataset was not available or reported by area or depth. The survey-derived mean *L_T_* showed significant variability among years (Welch’s test, *F* = 2.7, *p* = 0.004), with individuals captured in 2002–2003 showing smaller sizes than those captured in 1997 and 2013 (Bonferroni, *p* ≤ 0.047; Figure 5). From the official landings, the mean *L_T_* showed a significant (Welch’s test, *F* = 626.3, *p* < 0.001) decreasing pattern over the years (Bonferroni, *p* ≤ 0.041; Figure 5).

### 3.4. Growth Parameters

The size classes used in ELEFAN_GA ranged from 37.0 to 102.0 cm *L_T_* (Table S2). The estimated growth parameters are shown in Table 2 and Figure S1. The best fitted parameters obtained from *L_T_*–frequency data for the period 2010–2016 were *L_∞_* = 92.16 cm *L_T_*, *k* = 0.10 year^−1^, and *Φ* = 2.97 (Rn score = 0.69). Only 23 tagged skates (13 females, 5 males, and 5 unsexed) were considered for the growth analysis, as their time at liberty and their *L_T_* increment were properly recorded. Of these, individuals with *L_T_* increment equal to zero and time at liberty of less than 60 days were excluded from the dataset (Table S1). Thus, 20 skates (11 females, 5 males, and 4 unsexed) used in the final analysis produced estimates of *L_∞_* = 125.2 cm *L_T_* and *k* = 0.08 year^−1^ for combined sexes, *L_∞_* = 133.8 cm *L_T_* and *k* = 0.06 year^−1^ for females, and *L_∞_* = 21.8 cm *L_T_* and *k* = −0.15 year^−1^ for males (Table 2). However, estimates of *L_∞_* and *k* values using tag–recapture data showed poor adjustments (combined sexes: R^2^ = 0.08; females: R^2^ = 0.07; males: R^2^ = 0.01) and, therefore, results were not considered reliable. 

### 3.5. Sex Ratio

**Figure 6:**
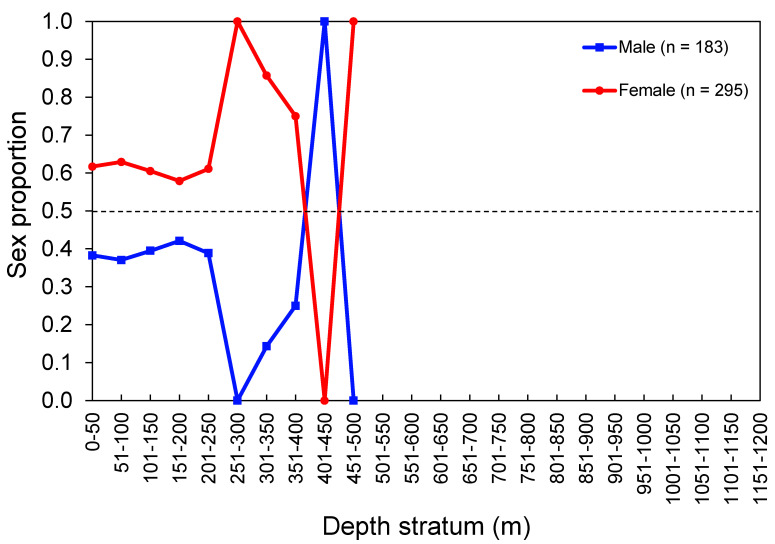
Sex proportion of the thornback ray *Raja clavata* by depth stratum. Data from the scientific surveys in the Azores. The number of individuals (n) refers to the total number of sampled thornback rays. The dashed line shows an equal sex ratio.

**Figure 7:**
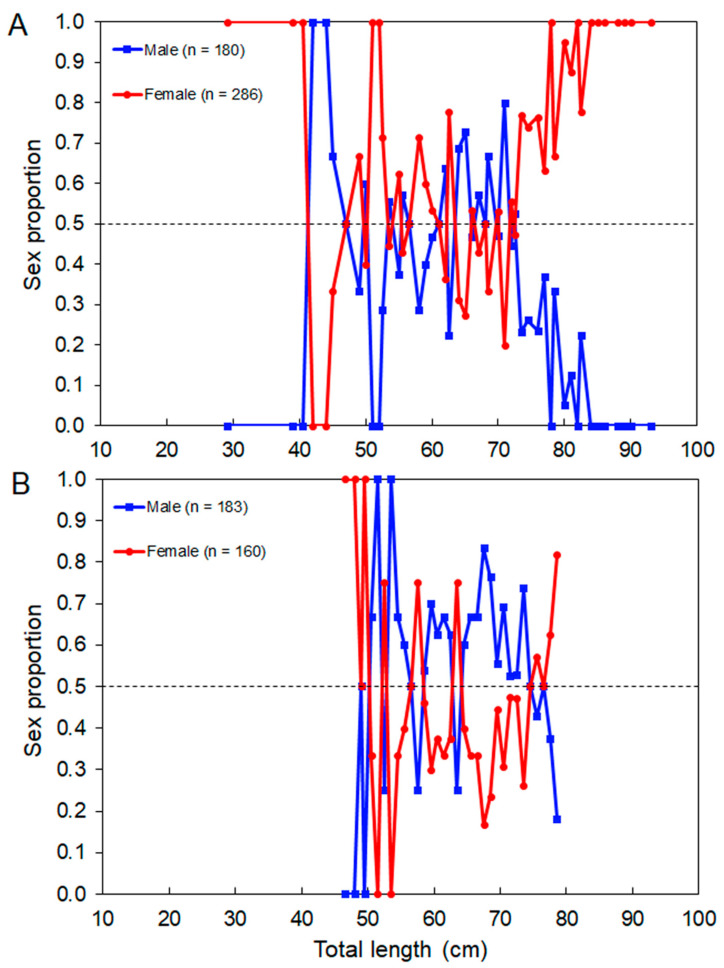
Sex proportion of the thornback ray *Raja clavata* by total length (*L_T_*)–class derived from the (**A**) scientific surveys and (**B**) commercial catches in the Azores. The number of individuals (n) refers to the total number of sampled thornback rays. The dashed line shows an equal sex ratio.

The sex ratio (M:F) observed in the whole surveyed area was 0.62:1, which departed from the expected 1:1 rate (χ^2^ = 26.24, *p* < 0.001). The overall sex ratio observed in the commercial catch samples (1.14:1) was not statistically different from 1:1 (χ^2^ = 1.54, *p* = 0.214). Females were significantly more abundant than males in island regions (χ^2^ = 25.08, *p* < 0.001) and in depths between 50 and 150 m (χ^2^ > 6.94, *p* < 0.008) and 250 and 300 m (χ^2^ > 5.00, *p* = 0.025; Figure 6). Sex-related information from the DCF dataset was not available or reported by area or depth. Males significantly dominated the *L_T_*–classes between 65 cm and 74 cm (surveys: χ^2^ > 7.20, *p* < 0. 007; commercial catches: χ^2^ > 4.000, *p* < 0.045); from this size, the sex ratio was inversed (surveys: χ^2^ > 4.000, *p* < 0.045; commercial catches: χ^2^ > 4.000, *p* < 0.045; Figure 7). The sex proportion equal to 0 or 1 in some depth strata and *L_T_*–classes was clearly driven by the low number of sampled individuals; therefore, this was not considered ecologically meaningful.

### 3.6. Reproduction

**Figure 8:**
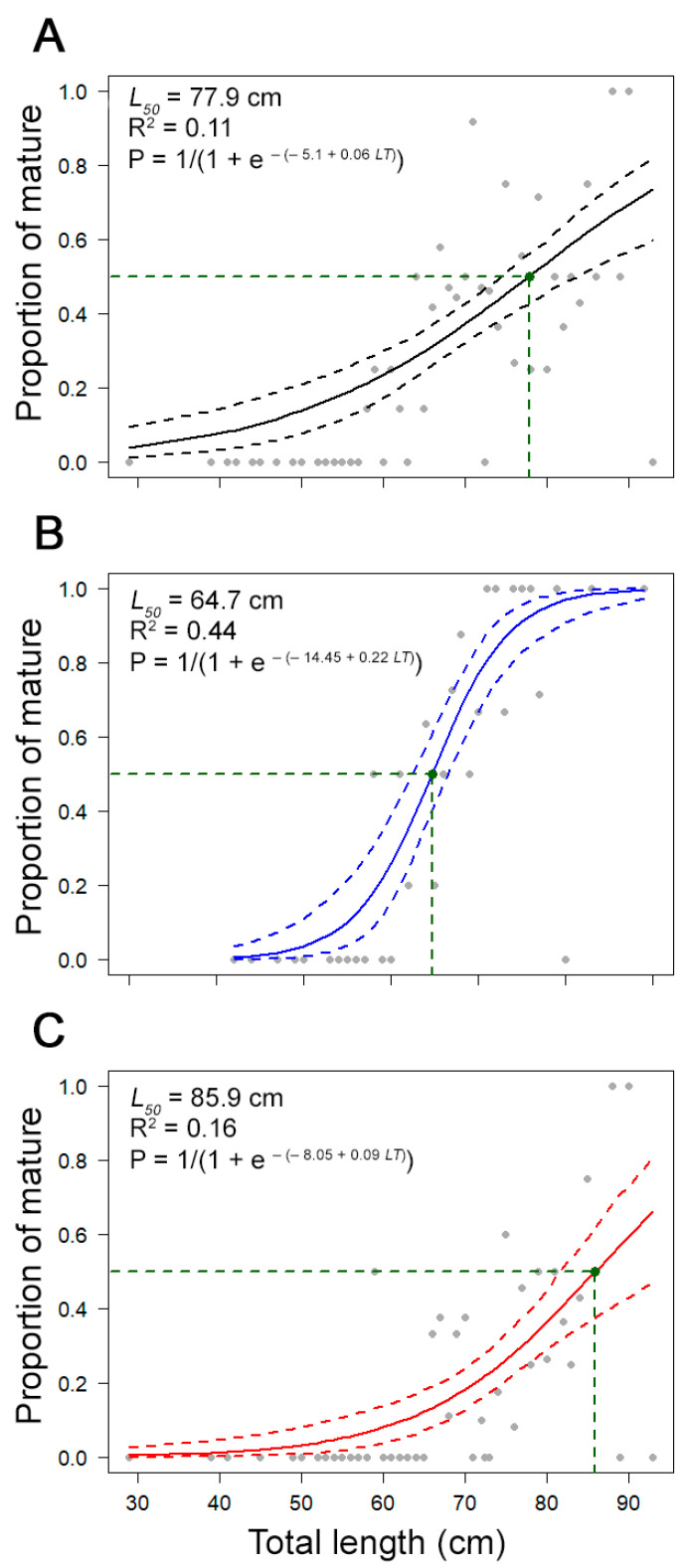
Maturity ogives for (**A**) combined sexes, (**B**) males, and (**C**) females of the thornback ray *Raja clavata* in the Azores. The solid curve represents the estimated logistic curve (±0.95 confidence intervals), and the dots represent the observed proportion of mature fish. Data from the scientific surveys.

A total of 155 individuals (115 females and 40 males) were considered as immature, 76 (60 females and 16 males) as developing, 52 (24 females and 28 males) as spawning capable, and 88 (30 females and 58 males) as actively spawning. The smallest mature female was observed at 59.0 cm *L_T_*, and the smallest mature male at 58.0 cm *L_T_.* Female’s maturity ogive presented high variability, particularly for individuals larger than 70 cm *L_T_*. The estimated *L_50_* was 85.9 cm *L_T_* for females (R^2^ value = 0.16), 64.7 cm *L_T_* for males (R^2^ value = 0.44), and 77.9 cm *L_T_* for combined sexes (R^2^ value = 0.11; Figure 8). For females and combined sexes, the diagnostic plots for the fitted models showed a relatively poor adjustment (low R^2^ value). 

### 3.7. Mortality, Exploitation Rate, and Size at Capture

Total mortality (*Z*), fishing mortality (*F*), and natural mortality (*M*) for the period 2010–2016 were estimated at 0.30 year^−1^, 0.14 year^−1^, and 0.16 year^−1^, respectively. The exploitation rate (*E*) was determined at 0.47. The *L_T_* at which 100% of individuals were vulnerable to capture (*L_c_*) was set at 67.0 cm. Details on the estimated values are shown in Table 2.

### 3.8. Catch Rates and Landings

**Figure 9:**
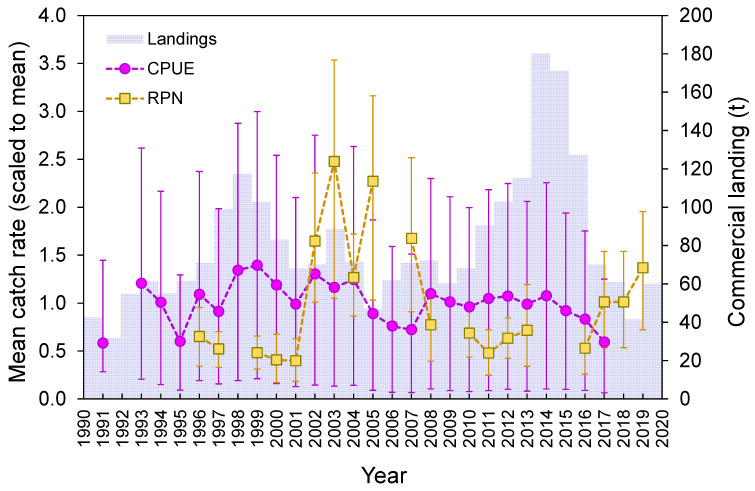
Official commercial landings (tons), mean (±0.95 confidence interval), standardized CPUE (kg days at sea^−1^ vessel^−1^), and mean (±0.95 confidence interval) survey-derived RPN (ind. 10^−3^ hooks) for the thornback ray *Raja clavata* in the Azores.

Despite the great interannual variability in the observed survey-derived abundance index (RPN), statistically non-significant interannual differences (Welch’s test, *F* = 1.3, *p* = 0.211; Figure 9) were detected. Standardized CPUE from the commercial fleet and official landings showed an oscillation over time, with a decreasing trend since 2014 (Figure 9). 

## 4. Discussion

Depth and temperature are often responsible for much of the spatial variation in the thornback ray *Raja clavata* (e.g., [9,71,72]), as well as in other demersal fish species (e.g., [73,74]). Temperature is a depth-related environmental factor important for fishes because it influences the rates of physiological processes, including metabolism and development [75]. This variable could boost habitat predictions, but it was not available at a fine-scale resolution in this study; when obtained from global datasets, it can have a poor predictive capacity [76]. In the absence of such data, depth is assumed to be the most important predictor variable of population density in studies of the spatial distribution of fish [76,77,78]. Around the Azores archipelago, *R. clavata* was more abundant in depths above 150 m, reflecting a spatial distribution restricted to coastal areas (98% of the total catch from the survey was around the islands; Figure 3), in which shallower depths are mostly available. The importance of depth is less clear than temperature, but it can be linked to other critical ecosystem variables such as prey concentration and bottom type [79].

Rajidae species are known to live on a variety of substrates, with sand or mud being the most common [72,80]. Although *R. clavata* has been registered throughout the Azores, mainly on coarse sediment and sandy mud bottoms, the EMODnet substrate layer was not available at a fine-scale resolution, and the results could be underestimated (i.e., the analyses could be influenced by a low number of observations for some factors). However, those soft bottoms were also the preferred substrate type of both captive and wild thornback rays inhabiting other regions [71,81,82,83]. This preference is partly attributed to the distribution of their preferred prey, as sand shrimps (e.g., *Solenocera membranacea*, *Crangon vulgaris*) are the most frequent food items in their stomachs [84,85]. Prey abundance is a significant and important limiting factor affecting predators’ abundance, and therefore defines habitat quality for some demersal fish species [86]. The thornback ray *R. clavata*, on the other hand, is a well-known opportunistic, mobile, and active predator, with a wide variety of prey [84,85,86,87]. Due to their large food ranges and mobility, these skates are likely to have a large foraging area, resulting in a mismatch between their abundance and that of their prey [86]. Prey abundance can thus have fewer limiting effects on the abundances of *R. clavata*; however, the influence of this parameter was beyond the reach of the current study and deserves further analysis.

Conventional tagging experiments on thornback rays in the Azores and Southern North Sea [87,88] have shown small-scale movements, with the majority of fish recaptured near the release point. Thornback rays are not, therefore, thought to have long-distance migrations similar to the winter skate *Leucoraja ocellata* [89]. However, it is widely acknowledged that evidence regarding fish movements and distribution obtained from release and recapture sites, time at liberty, and fishing effort estimates much more accurately describe the distribution of fishing fleets than the true level of fish dispersion [90,91]. Furthermore, since direct mortality caused by external tagging appears to be uncommon (most of the fish were in good condition at the time of release, and less active and inactive individuals were even recaptured; Table S1), the low recovery rate of *R. clavata* was most likely due to fishermen’s lack of cooperation in reporting the tags [92]. Therefore, the low tag reporting rate, along with inconsistent recapture location reporting from fishers, can make determining the species habitat range challenging [93]. This emphasizes the importance of collecting fishery-independent data while encouraging tag reporting to accurately describe true demographics.

Size-specific spatial segregation was not observed for *R. clavata* inhabiting islands and seamount areas of the Azores. On the other hand, segregation of sizes by depth in which larger individuals were found in deeper waters was observed; even the hook and line were not as effective for sampling small individuals (few skates under 40 cm were caught in this study; for example, Figure 3). The presence of smaller individuals in shallower waters forming aggregations has been related to the use of coastal areas for growth before moving offshore [88,94]. Contrary to the survey that primarily samples the areas around the islands [24], fishing efforts have been directed to more offshore habitats [9,74,95], which could justify a slightly greater frequency in large individuals in commercial landings. This latter point, on the other hand, is also highly influenced by market prices and restriction measures such as minimum landing size (MLS). For *R. clavata*, an MLS of 52 cm *L_T_* has been adopted in the Azores since 2015 [18]. This value appears to be close to the size at first maturity (e.g., [13,71,96]), but it is much smaller than the size at 50% estimated in this study and from the literature (see references below). Despite its susceptibility to fishing pressure, the impact of size-selective fishing or recent management measures on *R. clavata* size distribution may be hidden by its slow growth rate [9]. Therefore, the annual mean size patterns shown here for the Azorean region should be examined with caution.

Growth parameters observed in this study were consistent with those in the literature (Table S2) confirming that *R. clavata* is a long-living and slow-growing skate. However, these estimated parameters seem unreliable when they are compared with larger specimens (178 cm *L_T_*). This inconsistency could be related a sampling problem, as the occurrence of individuals larger than 95 cm *L_T_* seems rare (see Figure 3). The confirmation and elimination of suspected outliers, on the other hand, was not performed, since there was no scientific basis for this, as these specific results originated from sampling conducted more than 15 years ago. At the same time, it is highly recommended that growth parameters estimated from length–frequency analysis be further confirmed through readings of rigid structures because of the difficulty of detecting differences in age after determined sizes. The results from this study should, therefore, be used carefully.

The overall sex ratio was close to 1:1, which was consistent with previous results from other regions (e.g., [83,97]). However, a certain imbalance in this proportion can be understood as a result of different migration patterns between males and females [98]. This imbalance in the sex ratio favored males among the adults and females among the juveniles in the Bay of Douarnenez, Iroise Sea [98]. In the Azores, the population structure of *R. clavata* showed a favoring of females around the islands, which was reflected in the whole sample, since the scientific surveys essentially occur in these areas. As stated before, in this area, there was a slightly greater abundance of small individuals. However, the imbalance in sex ratio was not too evident in the smaller size classes (Figure 7), probably due to the aforementioned selectivity issue. Generally, male and female thornback rays develop at the same rate when they are young (1–4 years), but males’ growth rates tend to decline after four years [99]. In fact, the largest thornback rays are always female [71,100,101,102]. Differences in growth between the sexes may be due to maturation timing [103]. According to Walmsley-Hart et al. [104], female skates attain a larger size and grow slower as a result of their reproductive strategy; males mature faster to achieve sexual maturity, while females grow larger to hold the egg cases within the body cavity. 

In this study, 50% of female and male thornback rays were mature at 85.9 and 64.7 cm *L_T_*, respectively. According to Serra-Pereira et al. [13], females and males of *R. clavata* on the Portuguese continental shelf mature at 78.4 and 67.6 cm *L_T_*, respectively. Studies in other areas indicated that the *L_50_* ranged between 61.2 and 105.0 cm *L_T_* in females and between 58.8 and 82.3 cm *L_T_* in males [105]. Although the values estimated in this study were within these intervals, they must be interpreted with caution, particularly for females, given the high variability observed on the maturity ogive resulting in a poor adjustment (low R^2^). Considering that *R. clavata* matures late, at about 80% of its maximum size [13,99,106], and that the *L_∞_* was estimated in this study at 92.2 cm *L_T_*, a *L_50_* of 85.69 cm *L_T_* for females might in fact be overestimated. Therefore, it is increasingly suggested to review the onboard biological survey sampling methods implementing a maturity scale more adapted to the resource (for example, including the post-spawning stages), and running this sampling more systematically (for example, on an annual basis) to overcome these issues. 

Estimates of thornback ray spawning season often vary across geographical areas, as well as within the same region [13]. Spawning in UK coastal waters, for example, was estimated to take place between February and September, with a peak in June [107,108]; other authors indicated a later start to spawning (March or May) [96,109]. In Southern areas, spawning seasons are longer, extending from May to December (Black Sea), or even occurring all year (Northwest Mediterranean) [103,110,111]. In Portuguese continental waters, spawning was also found throughout the year, but the proportion of females in spawning condition was higher between May and January, with a peak in August [13]. A similar reproductive strategy may occur in the Azores; however, available data were not sufficient to confirm it. Some specific technical considerations have not always been considered in current Azorean data collection programs, and collected data cannot meet the necessary precision levels for some species. In some cases, for example, the species’ spawning season does not coincide with the sampling time, as is the case of the Azorean spring bottom longline survey; in others, the sampling does not cover all habitats of the species, as is the case of the fishery-dependent data that come mainly from offshore areas due to fishing area restrictions. Furthermore, the gonad maturity scales in use, as well as the understanding of specific stages (e.g., immature versus post-spawning), contribute to misclassifications, resulting in an inaccurate estimate of spawning stock biomass [112]. This highlights the need for species-specific long-term research and the validation of macroscopic staging by histology [113].

Overfishing of *R. clavata* has been detected in other parts of the Atlantic [96,99,114,115] and Mediterranean [83,116]. In the Azores, historical trends of commercial catch rates and landings are frequently marked by temporal changes in the market demand and management measures, particularly catch limits (TAC/quotas) [9]. Thus, the reduction in landings and commercial catches of *R. clavata* observed in recent years may be more associated with an increase in discards than a decline in the abundance of the stock [9]. Survey-derived abundance indices represent an unbiased accounting of healthy stock, since they are not influenced by these factors. On the other hand, environmental characteristics, such as the substrate type, can greatly affect the abundance indices [9,24] and generate the oscillatory pattern observed in the present study. Since abundance indices are often used as a key input parameter in fish stock assessment models [117], using statistical approaches to minimize the impact of complex variables is critical. 

Given that the exploitation rate (*E*) estimated for the most recent 2010–2016 period (*E* = 0.47) was below the optimal level of 0.50 and fishing mortality (*F*) was lower than natural mortality (*M*) [67], there is no clear evidence that this species is overexploited in the Azorean region. However, considering that growth parameters, despite being adjusted to the data, did not fit to biology (*L_max_* = 178 cm *L_T_* versus *L_∞_* = 92.2 cm *L_T_*), the scenario could be much worse than *E* = 0.47. Lower *k* and higher *L_∞_* would lead to a lower natural mortality and, consequently, higher *F* that implies higher *E*. Therefore, as *R. clavata* is a commercially important elasmobranch species, improving data quality and input information for analytical stock assessment should be a priority. In this regard, estimations of additional biological and fishery parameters, such as length at first maturity (*L_m_*), length at maximum possible yield (*L_opt_*), life span (*t_max_*), and theoretical age at length zero (*t_0_*), were performed by using the estimated growth parameters (*L_∞_* and *k*) as input of some empirical equations (Table S3) [118,119]. As well as other deep-water species [18,92,95,120,121,122,123], *R. clavata* was characterized by the k-selected life history strategy with large size, slow growth, low natural mortality (Table 2), long life span, and late maturity (Table S1). The empirical equations also suggested a healthy fished population in the Azores, with the *L_c_* (Table S3) above the *L_m_* and *L_opt_* (Table S1), and the mean *L_T_* in the catch (Figure 5) above the *L_c_*, *L_m_,* and *L_opt_*. It should be emphasized that the approximate numerical estimates are preliminary and should be interpreted with caution, since the consequences of an underestimated *L_∞_* would result in a more concerning situation. The findings must therefore be checked first (e.g., [124,125]), and only then used for management before specific evidence becomes accessible. 

## 5. Conclusions

*Raja clavata* is a near threatened elasmobranch species and, while our findings suggested a relatively healthy stock in the Azores, a substantial improvement in data quality is necessary to better understand the stock condition and prevent overexploitation. Reliable management strategies require actual knowledge about habitat preferences, vertical distribution, movement pattern, size–frequency distributions, growth parameters, sex ratios, mortality rates, and reproduction of this species. This study expands current knowledge on the thornback ray *R. clavata* population inhabiting the Azorean region and provides a baseline for further monitoring and comparative studies. However, further studies (e.g., reliability of the abundance indices from the survey, growth parameters estimate from direct readings, reproductive aspects, and habitat prediction using in situ substrate data) are recommended to make additional advances in stock characterization. Finally, using data-limited approaches, the stock size and biological reference points should be assessed in order to achieve the highest sustainable yield.

## Figures and Tables

**Table 1:** Results of the generalized additive models for the thornback ray *Raja clavata* abundances derived from the scientific surveys (1996–2019) in the Azores. Bottom type: coarse sediment (C. Sed, termed as Intercept), mixed sediment (Mix.Sed), mud (Mud), muddy sand (Mud.S), rock (Rock), sand (Sand), and sandy mud (Sand.M).

Family	Link Function		Formula				Adjusted R^2^		Deviance Explained
Binomial	logit		RPN.Bi ~ s(Longitude, Latitude) + s(Depth, k = 4) + Substrate				0.283		37.86%
Gaussian	identity		RPN ~ s(Longitude, Latitude) + s(Depth, k = 4) + Substrate				0.137		16.30%
**Binomial**					**Gaussian**				
**Parametric coefficients**									
	**Estimate**	**Std. Error**	**z value**	**Pr(>|z|)**		**Estimate**	**Std. Error**	**z value**	**Pr(>|z|)**
(Intercept)	−5.086	0.264	−19.233	<0.001	(Intercept)	0.721	0.198	3.648	<0.001
SubstrateMix.Sed	−0.014	0.194	−0.071	0.944	SubstrateMix.Sed	0.173	0.216	0.798	0.425
SubstrateMud	0.423	0.879	0.481	0.631	SubstrateMud	−0.346	1.063	−0.325	0.745
SubstrateMud.S	−0.546	0.621	−0.879	0.380	SubstrateMud.S	−0.468	0.769	−0.609	0.543
SubstrateRock	−0.087	0.209	−0.415	0.678	SubstrateRock	−0.196	0.235	−0.834	0.405
SubstrateSand	0.214	0.215	0.999	0.318	SubstrateSand	0.330	0.238	1.386	0.166
SubstrateSand.M	4.657	1.220	3.817	<0.001	SubstrateSand.M	−0.917	1.502	−0.611	0.542
**Binomial**					**Gaussian**				
**Approximate significance of smooth terms**									
	**edf**	**Ref. df**	**Chi. sq**	***p*** **-value**		**edf**	**Ref. df**	**Chi. sq**	***p*-value**
s (longitude, latitude)	27.712	28.830	340.200	<0.001	s (Longitude, Latitude)	14.490	18.563	3.104	<0.001
S (depth)	1.344	1.580	278.200	<0.001	s (Depth)	2.670	2.917	8.677	<0.001

**Table 2:** Growth and fishery parameters for the thornback ray *Raja clavata* in the Azores estimated from *L_T_*–frequency data for the period 2010–2016. DCF: data from the EU Data Collection Framework. F + M: combined sexes. F: females. M: males. Lower and upper denote (a) 95% confidence interval, (b) standard deviation, or (c) standard error limits of the estimates.

Parameters	Input Data	Method	Sex	Estimates	Lower	Upper
Asymptotic length (*L_∞_*; cm *L_T_*)	DCF	ELEFAN_GA_Boot [44]	F + M	92.16	90.22 ^a^	94.76 ^a^
	Tag–recapture	[48]	F + M	125.21	–	–
	Tag–recapture	[48]	F	133.83	–	–
	Tag–recapture	[48]	M	21.76	–	–
Growth coefficient (*k*; year^−1^)	DCF	ELEFAN_GA_Boot [44]	F + M	0.104	0.099 ^a^	0.128 ^a^
	Tag–recapture	[48]	F + M	0.08	−0.05 ^a^	0.21 ^a^
	Tag–recapture	[48]	F	0.06	−0.11 ^a^	0.24 ^a^
	Tag–recapture	[48]	M	−0.15	−3.13 ^a^	2.83 ^a^
Growth performance index (*Φ*)	DCF	ELEFAN_GA_Boot [44]	F + M	2.97	2.92 ^a^	3.04 ^a^
Natural mortality (*M*; year^−1^)	–	[53]	F + M	0.18	–	–
	–	[54]	F + M	0.17	–	–
	–	[59]	F + M	0.10	–	–
	–	[60]	F + M	0.10	–	–
	–	[61]	F + M	0.26	–	–
	–	[62]	F + M	0.24	–	–
	–	[63]	F + M	0.10	–	–
	–	[64]	F + M	0.15	–	–
	–	[65]	F + M	0.20	–	–
	–	[58]	F + M	0.15	–	–
	–	[66]	F + M	0.17	–	–
	–	[66]	F + M	0.16	–	–
	–	[55]	F + M	0.15	–	–
	–	[56]	F + M	0.14	–	–
	–	[57]	F + M	0.14	–	–
	–	Average *M* value	F + M	0.16	0.11 ^b^	0.21 ^b^
Total mortality (*Z*; year^−1^)	DCF	[51]	F + M	0.30	0.29 ^c^	0.31 ^c^
	DCF	[52]	F + M	0.30	0.27 ^a^	0.32 ^a^
Fishing mortality (*F*; year^−1^)	–	*F = Z − M*	F + M	0.14	–	–
Exploitation rate (*E*)	–	[67]	F + M	0.47	–	–
Length of full selectivity (*L_c_*; cm *L_T_*)	DCF	[51]	F + M	67.0	–	–

## Data Availability

Data supporting reported results are available from the corresponding author upon reasonable request.

## Supplementary Materials

The following are available online at https://www.mdpi.com/article/10.3390/biology10070676/s1, Figure S1. Growth curves (dashed lines) for *Raja clavata* in the Azores plotted through the *L_T_*–frequency data obtained using bootstrapped ELEFAN_GA model. Black bars indicate positive values (peaks), whereas white bars indicate negative peaks. Shading refers to the difference between moving averages. Data from the EU Data Collection Framework (DCF) for the period 2010–2016. Figure S2. Monthly mean (±0.95 confidence intervals) gonadosomatic index (GSI) and hepatosomatic index (HSI) for the thornback ray *Raja clavata* in the Azores. Data from the commercial catches. Table S1. Summary of the number of tagged and recaptured thornback rays *Raja clavata* in the Azores. Distance traveled was measured by tracing a straight line between capture and recapture geographical positions. *L_T_*1: total length (*L_T_*, cm) at capture. *L_T_*2: *L_T_* at recapture Δ*L_T_*: the difference between *L_T_*1 and *L_T_*2. Δ*T*: time at liberty expressed in days. Δ*L_T_* year^−1^: the annual *L_T_* increment. Bold highlights the 20 selected recaptures used in the growth analysis (Δ*L_T_* larger than zero and Δ*T* larger than 60 days). Table S2. Growth parameters estimated by other authors for *Raja clavata* in European waters. F: female; M: male; n: number of individuals; *L_∞_*: the asymptotic length; *k*: the growth coefficient; *t_0_*: the theoretical age at length zero. Table S3. Estimates of biological and fishery parameters for *Raja clavata* calculated from the empirical relationships between the length at first maturity (*L_m_*), length at maximum possible yield (*L_opt_*), life span (*t_max_*), and theoretical age at length zero (*t_0_*), and the asymptotic length (*L_∞_*) and growth coefficient (*k*). The values of *L_∞_* and *k* were derived from the *L_T_*–frequency data collected for the period 2010–2016 as part of the EU Data Collection Framework (DCF).
