# Supplementary material for: Biological Knowledge of Thornback Ray (Raja clavata) from the Azores: Improving Scientific Information for the Effectiveness of Species-Specific Management Measures
Source: Biology (Basel). 2021 Jul 17;10(7):676. doi: 10.3390/biology10070676 (PMC8301307; doi:10.3390/biology10070676)
Supplement: Supplementary file 1 [file biology-10-00676-s001.zip › biology-1302311-supplementary.pdf]

Biological Knowledge of Thornback Ray (*Raja clavata*) from the Azores: Improving Scientific Information for the Effectiveness of Species-Specific Management Measures

Régis Santos [1,2,*], Wendell Medeiros-Leal [1,2], Ana Novoa-Pabon [2,3], Osman Crespo [2] and Mário Pinho [1,2,3]

[1] IMAR Institute of Marine Research, University of the Azores, 9901-862 Horta, Portugal; wendell.mm.silva@uac.pt (W.M.-L.); mario.rr.pinho@uac.pt (M.P.)

[2] Okeanos R&D Centre, University of the Azores, 9901-862 Horta, Portugal; osman.cresponeto@imbrsea.eu (O.C.); ana.mn.pabon@uac.pt (A.N.-P.)

[3] Department Oceanography and Fisheries, Faculty of Science and Technology, University of the Azores, 9901-862 Horta, Portugal

* Correspondence: regis.vs.santos@uac.pt; Tel.: +351-292200469

Supplementary material

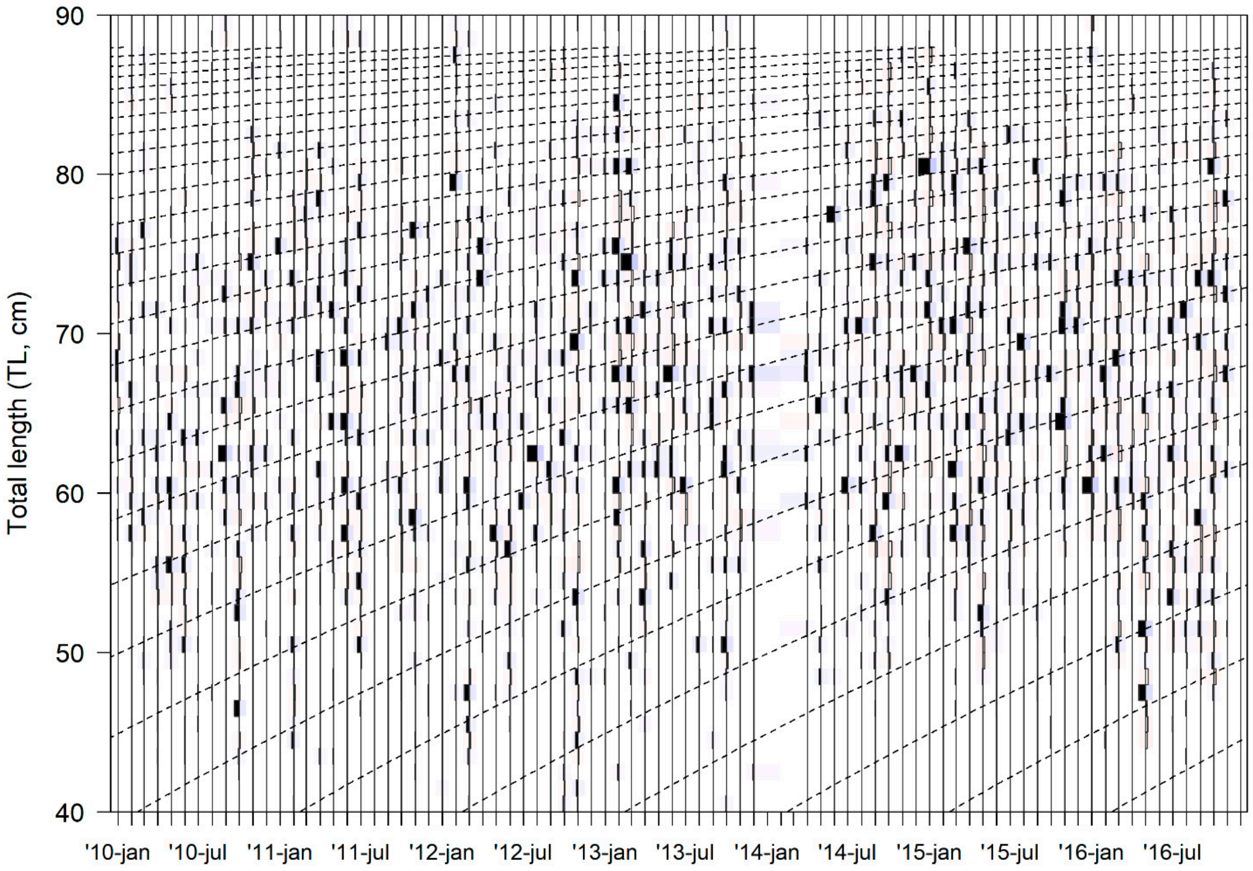

Figure S1. Growth curves (dashed lines) for *Raja clavata* in the Azores plotted through the L_T–frequency data obtained using bootstrapped ELEFAN_GA model. Black bars indicate positive values (peaks), whereas white bars indicate negative peaks. Shading refers to the difference between moving averages. Data from the EU Data Collection Framework (DCF) for the period 2010–2016.

Table S1. Summary of the number of tagged and recaptured thornback rays *Raja clavata* in the Azores. Distance traveled was measured by tracing a straight line between capture and recapture geographical positions. L_T1: total length (L_T, cm) at capture. L_T2**:** L_T at recapture ΔL_T: the difference between L_T1 and L_T2. ΔT: time at liberty expressed in days. ΔL_T year^{-1}: the annual L_T increment. Bold highlights the 20 selected recaptures used in the growth analysis (ΔL_T larger than zero and ΔT larger than 60 days).

No.	Sex	Fish condition at release	Distance travelled (km)	L_T1 (cm)	L_T2 (cm)	ΔL_T (cm)	ΔT (days)	ΔL_T year^{-1}
1	–	Active	–	61.0	–	–	291	–
**2**	**F**	Active	**37.4**	**63.0**	**71.0**	**8.0**	**325**	**8.985**
3	F	Active	–	76.0	–	–	426	–
4	F	Active	35.3	81.0	81.0	0.0	15	0.000
**5**	–	Active	**37.2**	**37.0**	**85.5**	**48.5**	**1864**	**9.497**
**6**	**M**	Active	–	**49.0**	**54.0**	**5.0**	**491**	**3.717**
**7**	**F**	Active	–	**49.0**	**72.0**	**23.0**	**1548**	**5.423**
**8**	**F**	Active	**40.5**	**50.0**	**73.0**	**23.0**	**1202**	**6.984**
**9**	**F**	Active	–	**51.0**	**59.0**	**8.0**	**481**	**6.071**
**10**	**F**	Active	–	**53.0**	**58.0**	**5.0**	**1285**	**1.420**
**11**	**M**	Active	–	**55.0**	**59.0**	**4.0**	**110**	**13.273**
12	M	Active	–	56.0	–	–	397	–
13	F	Active	–	56.0	–	–	397	–
**14**	**M**	Active	–	**56.0**	**60.0**	**4.0**	**324**	**4.506**
**15**	**M**	Active	–	**57.0**	**60.0**	**3.0**	**278**	**3.939**
16	–	Active	–	57.5	50.0	–	868	–
17	F	Active	–	59.0	–	–	316	–
18	F	Active	–	63.0	–	–	472	–
**19**	**F**	Active	–	**63.0**	**66.0**	**3.0**	**325**	**3.369**
20	F	Active	9.1	69.0	69.5	0.5	11	16.591
**21**	**F**	Active	**37.3**	**71.0**	**71.5**	**0.5**	**123**	**1.484**
**22**	**F**	Active	**35.1**	**76.0**	**84.4**	**8.4**	**524**	**5.851**
**23**	**F**	Active	**34.1**	**77.0**	**81.0**	**4.0**	**619**	**2.359**
**24**	–	Active	**10.5**	**80.0**	**83.0**	**3.0**	**244**	**4.488**
**25**	**F**	Active	**38.6**	**80.0**	**85.0**	**5.0**	**930**	**1.962**
**26**	–	Active	**33.8**	**85.0**	**88.0**	**3.0**	**556**	**1.969**
27	–	Less Active	37.5	53.0	53.0	0.0	66	0.000
**28**	**M**	Less Active	–	**56.5**	**58.0**	**1.5**	**455**	**1.203**
**29**	–	Less Active	**12.2**	**69.0**	**80.0**	**11.0**	**1032**	**3.891**
30	F	Less Active	–	44.0	–	–	175	–
**31**	**F**	Less Active	–	**63.0**	**68.0**	**5.0**	**283**	**6.449**
32	F	–	–	55.5	–	–	1913	–
33	F	Active	–	68.0	74.0	6.0	–	–
34	M	Active	–	71.0	72.0	1.0	–	–
35	F	Inactive	–	48.0	62.0	14.0	–	–
Mean		–	30.7	61.7	69.6	7.6	573.3	4.9
± s.d.		–	11.6	11.7	11.3	10.3	506.0	4.1

Table S2. Growth parameters estimated by other authors for *Raja clavata* in European waters. F: female; M: male; n: number of individuals; L_∞: the asymptotic length; *k*: the growth coefficient; t_0: the theoretical age at length zero.

Geographical region	Sex	Length range (cm)	n	L_∞	*k*	t_0	Input data	Reference
North Sea	F	–	51	118.0	0.14	-0.88	Vertebrae	[1]
North Sea	M	–	41	98.0	0.17	-0.43	Vertebrae	[1]
Irish Sea	F + M	9.0–107.0	2918	105.0	0.22	0.45	Length-frequency analysis	[2]
Irish Sea	F	–	1504	107.8–120.0	0.15–0.26	-1.01–0.05	Vertebrae	[3]
Irish Sea	M	–	783	96.8–104.3	0.19–0.24	-1.36–0.32	Vertebrae	[3]
Irish Sea	M	12.0–96.5	165	106.5	0.14	-1.74	Vertebrae	[4]
Irish Sea	F	13.1–105.5	93	139.5	0.09	-1.84	Vertebrae	[4]
Irish Sea and Bristol Channel	F	30.0–89.0	64	128.1	0.09	-1.32	Tag–recapture	[5]
Irish Sea and Bristol Channel	M	25.0–89.0	57	85.6	0.21	-0.60	Tag–recapture	[5]
Carmarthen Bay, UK	F + M	13.0–99.0	2143	139.2	0.09	-2.63	Vertebrae	[6]
Caernarfon Bay, UK	F	–	135	117.6	0.16	-0.71	Vertebrae	[7]
Caernarfon Bay, UK	M	–	54	100.9	0.18	-0.95	Vertebrae	[7]
United Kingdom	F	–	85	127.3	0.10	-2.50	Vertebrae	[8]
United Kingdom	M	–	61	88.3	0.22	-1.30	Vertebrae	[8]
Portuguese Continental Coast	F + M	13.0–89.0	200	130.5	0.10	-0.14	Dermal denticles	[9]
Portuguese Continental Coast	F	13.0–89.0	115	130.5	0.10	-0.13	Dermal denticles	[9]
Portuguese Continental Coast	M	15.0–77.0	85	121.5	0.11	-0.11	Dermal denticles	[9]
Portuguese continental coast	F + M	19.5–91.3	251	96.7–128.0	0.12	-0.62–0.58	Dermal denticles	[10]
Portuguese continental coast	F	19.9–91.3	129	101.4–140.7	0.1	-0.88–0.50	Dermal denticles	[10]
Portuguese continental coast	M	19.5–87.0	122	90.6–117.1	0.14	-0.36–0.72	Dermal denticles	[10]
Mediterranean Sea	F	–	–	126.5	0.10	-0.51	Vertebrae	[11]
Mediterranean Sea	M	–	–	116.7	0.11	-0.41	Vertebrae	[11]
Mediterranean Sea	F	29.3–64.6	37	95.7	0.09	-2.60	Vertebrae	[12]
Mediterranean Sea	M	35.9–57.8	53	61.9	0.22	-1.79	Vertebrae	[12]
Mediterranean Sea	F + M	29.3–64.6	90	79.7	0.13	-2.70	Vertebrae	[12]
Mediterranean Sea	F + M	8.0–88.0	–	71.7–84.2	–	–	Length-frequency analysis	[13]
Mediterranean Sea	F	14.0–110.0	160	98.7–114.6	0.11–2.30	1.02–1.23	Vertebrae	[14]
Mediterranean Sea	M	14.6–89.0	125	92.3–100.8	0.14–0.40	0.95–1.13	Vertebrae	[14]
Azores	F + M	37.0–102.0	6963	92.2	0.10	0.78	Length-frequency analysis	Present study

Table S3. Estimates of biological and fishery parameters for *Raja clavata* calculated from the empirical relationships between the length at first maturity (L_m), length at maximum possible yield (L_{opt}), life span (t_{max}), and theoretical age at length zero (t_0), and the asymptotic length (L_∞) and growth coefficient (k). The values of L_∞ and k were derived from the L_T–frequency data collected for the period 2010–2016 as part of the EU Data Collection Framework (DCF).

**Parameter**	**Estimates**	**Empirical formula**	**Reference**
Length at first maturity (L_m)	48.50 cm L_T	$\log(L_m) = 0.8979 \times \log(L_\infty) - 0.0782$	[15]
Length at maximum possible yield (L_{opt})	59.30 cm L_T	$\log(L_{opt}) = 1.053 \times \log(L_m) - 0.0565$	[15]
Life span (t_{max})	30.00 years	$t_{max} = 3 / k$	[15]
Theoretical age at length zero (t_0)	0.78 years	$\log(-t_0) = -0.3922 - 0.2752 \times \log(L_\infty) - 1.038 \times \log(k)$	[16]

References

1. Walker, P.A. Fleeting images: dynamics of North Sea ray populations, Universiteit van Amsterdam [Host], 1999.
2. Brander, K.; Palmer, D. Growth rate of Raia clavata in the Northeast Irish Sea . *ICES J. Mar. Sci.* **1985**, *42*, 125–128, doi:10.1093/icesjms/42.2.125.
3. Fahy, E. The south eastern ray Raja spp. fishery, with observations on the growth of rays in Irish waters and their commercial grading. 1991.
4. Gallagher, M.J.; Nolan, C.P.; Jeal, F. Age, Growth and Maturity of the Commercial Ray Species from the Irish Sea. *J. Northwest Atl. Fish. Sci.* **2004**, *35*, 47–66, doi:10.2960/j.v35.m527.
5. Holden, M.J. The Growth Rates of Raja Brachyura, R. Clavata and R. Montagui as Determined From Tagging Data. *ICES J. Mar. Sci.* **1972**, *34*, 161–168, doi:10.1093/icesjms/34.2.161.
6. Ryland, J.S.; Ajayi, T.O. Growth and population dynamics of three Raja species (Batoidei) in Carmarthen Bay, British Isles. *ICES J. Mar. Sci.* **1984**, *41*, 111–120, doi:10.1093/icesjms/41.2.111.
7. Whittamore, J.M.; McCarthy, I.D. The population biology of the thornback ray, Raja clavata in Caernarfon Bay, north Wales. *Mar. Biol. Assoc. United Kingdom. J. Mar. Biol. Assoc. United Kingdom* **2005**, *85*, 1089.
8. Taylor, A.J.; Holden, M.J. The preparation and use of vertebrae for age determination in rays. *ICES C.* **1964**, *145*, 1–3.
9. Serra-Pereira, B.; Figueiredo, I.; Bordalo-Machado, P.; Farias, I.; Moura, T.; Gordo, L.S. Age and growth of Raja clavata Linnaeus, 1758--evaluation of ageing precision using different types of caudal denticles. *ICES Doc. C.* **2005**, 1–10.
10. Serra-Pereira, B.; Figueiredo, I.; Farias, I.; Moura, T.; Gordo, L.S. Description of dermal denticles from the caudal region of Raja clavata and their use for the estimation of age and growth. *ICES J. Mar. Sci.* **2008**, *65*, 1701–1709.
11. Ragonese, S.; Andreoli, M.G.; Bono, G.; Giusto, G.B.; Rizzo, P.; Sinacori, G. Overview of the available biological information on demersal resources of the Strait of Sicily. *MedSudMed Tech. Doc.* **2004**, *2*, 67–74.
12. Yeldan, H.; Avşar, D.; Manaşırlı, M. Some biological peculiarities of thornback ray, Raja clavata (Linnaeus, 1758) in the northeast Mediterranean. *Su Ürünleri Derg.* **2008**, *25*.

13. Bottari, T.; Rinelli, P.; Bianchini, M.L.; Ragonese, S. Stock identification of Rajaclavata L. (Chondrichthyes, Rajidae) in two contiguous areas of the Mediterranean. *Hydrobiologia* **2013**, *703*, 215–224, doi:10.1007/s10750-012-1361-0.

14. Kadri, H.; Marouani, S.; Saïdi, B.; Bradai, M.N.; Bouaïn, A.; Morize, E. Age, growth, sexual maturity and reproduction of the thornback ray, Raja clavata (L.), of the Gulf of Gabès (south-central Mediterranean Sea). *Mar. Biol. Res.* **2014**, *10*, 416–425, doi:10.1080/17451000.2013.797584.

15. Froese, R.; Binohlan, C. Empirical relationships to estimate asymptotic length, length at first maturity and length at maximum yield per recruit in fishes, with a simple method to evaluate length frequency data. *J. Fish Biol.* **2000**, *56*, 758–773, doi:https://doi.org/10.1111/j.1095-8649.2000.tb00870.x.

16. Pauly, D. *Theory and Management of Tropical Multi-Species Stocks: A Review, with Emphasis on the Southeast Asian Demersal Fisheries.*; International Center for Living Aquatic Resources Management: Manila, Philippines, 1979; Vol. 1;.
